# Supplementary material for: Strain- and age-dependent divergence in mouse appetitive spatial learning and decision strategies
Source: Front Behav Neurosci. 2026 Jun 23;20:1858691. doi: 10.3389/fnbeh.2026.1858691 (PMC13337451; doi:10.3389/fnbeh.2026.1858691)
Supplement: Supplementary file 1 [file Table_1.PDF]

Supplementary Table 1. Sample-size breakdown by strain, age, sex, and reward side

N indicates the number of animals included in each subgroup.

A. Sample size by strain and age

Strain	Age	N
C57BL/6	Younger	16
C57BL/6	Older	12
CBA/CaOlaHsd	Younger	14
CBA/CaOlaHsd	Older	12

B. Sample size by strain, age, and sex

Strain	Age	Sex	N
C57BL/6	Younger	Male	10
C57BL/6	Younger	Female	6
C57BL/6	Older	Male	12
C57BL/6	Older	Female	0
CBA/CaOlaHsd	Younger	Male	14
CBA/CaOlaHsd	Younger	Female	0
CBA/CaOlaHsd	Older	Male	7
CBA/CaOlaHsd	Older	Female	5

C. Sample size by strain, age, sex, and rewarded side

Strain	Age	Sex	Reward side	N
C57BL/6	Younger	Male	Left	3
C57BL/6	Younger	Female	Left	0
C57BL/6	Younger	Male	Right	7
C57BL/6	Younger	Female	Right	6
C57BL/6	Older	Male	Left	6
C57BL/6	Older	Female	Left	0
C57BL/6	Older	Male	Right	6
C57BL/6	Older	Female	Right	0
CBA/CaOlaHsd	Younger	Male	Left	6
CBA/CaOlaHsd	Younger	Female	Left	0
CBA/CaOlaHsd	Younger	Male	Right	8
CBA/CaOlaHsd	Younger	Female	Right	0
CBA/CaOlaHsd	Older	Male	Left	3
CBA/CaOlaHsd	Older	Female	Left	2
CBA/CaOlaHsd	Older	Male	Right	4
CBA/CaOlaHsd	Older	Female	Right	3
